# Evaluation of a user‐friendly CSDS cage apparatus for studying depressive‐like behaviors in rodents

**DOI:** 10.1002/ame2.12510

**Published:** 2024-12-03

**Authors:** Hao Zhang, Dongmei Gao, Minghu Hu, Wanqing Zhou, Muxuan Han, Ya Sun, Yang Zhang, Jieqiong Wang, Mingzhou Gao

**Affiliations:** ^1^ High‐Level Key Disciplines of Traditional Chinese Medicine: Basic Theory of Traditional Chinese Medicine, National Administration of Traditional Chinese Medicine Shandong University of Traditional Chinese Medicine Jinan China; ^2^ Key Laboratory of Traditional Chinese Medicine Classical Theory Ministry of Education, Shandong University of Traditional Chinese Medicine Jinan China; ^3^ Experimental Center Shandong University of Traditional Chinese Medicine Jinan Shandong China; ^4^ College of Traditional Chinese Medicine Shandong University of Traditional Chinese Medicine Jinan Shandong China; ^5^ Innovative Institute of Chinese Medicine and Pharmacy Shandong University of Traditional Chinese Medicine Jinan Shandong China; ^6^ Social Cooperation and Achievement Transformation Department Shandong University of Traditional Chinese Medicine Jinan Shandong China

**Keywords:** CSDS, depression, experimental cage, leak‐proof water bottle, methodology

## Abstract

**Background:**

Previously, a chronic social defeat stress (CSDS) model has been widely‐adopted for assessing depressive‐like behaviors in animals. However, there is still room for improvement in the CSDS model to safeguard study accuracy and the welfare of lab rodents. Our study team developed a novel, standardized apparatus to induce CSDS in rodents and assessed the model's practical adaptability.

**Methods:**

An innovative CSDS cage apparatus and water bottle was designed. To evaluate the effectiveness of the newly developed tools, a variety of animal models, including the tail suspension test (TST), sucrose preference test, forced swimming test (FST), novelty‐suppressed feeding test, female urine sniffing test, and open field test (OFT), were adopted to assess depressive‐like behaviors in mice. Fluoxetine treatment was also administered to observe the reversal effect, as part of the validation.

**Results:**

The CSDS cage apparatus resulted in the manifestation of depressive‐like behaviors in the model mice. Significant reductions in sucrose preference and urine sniffing time were observed, while the OFT revealed decreased central zone total distance, residence time, and frequency of entry. Moreover, increased immobility was found in the FST and TST. Fluoxetine treatment was found to successfully reverse the modeling effect.

**Conclusion:**

The CSDS cage apparatus was validated for enhanced usability and addressed the previous challenges of water bottle leakage and lab rodent welfare issues. The consistent results from multiple behavioral tests also supported real‐world application of the apparatus, offering researchers a promising alternative to conventional rodent cages.

## Graphical abstract

Using chronic social defeat stress cage apparatus, the model mice exhibited characteristic behaviors resembling depression, including a significant decrease in preference for sugar‐water, a notable reduction in the time spent sniffing female urine, a prolonged delay in initiating feeding, a substantial decrease in total distance traveled, residence time, and entries into the central zone in open field test, a significant decrease in the time taken to become immobile in forced swimming test (FST), a considerable increase in immobility time and the number of instances of immobility in FST and tail suspension test. These aforementioned behavioral manifestations were found to be reversible through drug intervention.

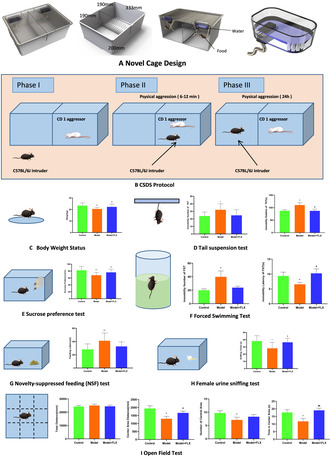

## 1 INTRODUCTION

Depression is the third leading component of the global disease burden, with a significant socioeconomic cost globally, complicated by high levels of morbidity, disability, and relapse. The major clinical manifestation include a loss of pleasure, reduced volition, slowed thinking, indifference to external stimulation, and suicidal behaviors.^1^, ^2^, ^3^ In 2018, the World Health Organization ranked depression as the third leading component of the global disease burden, and it is expected to top the list by 2030.^4^ Practically, the clinical detection, diagnosis, and management of the disease often pose challenges for clinicians due to its highly variable clinical manifestation, recurrent course, unpredictable prognosis, and differential response to treatment.^5^, ^6^

In recent years, a multitude of research efforts have been dedicated to the development of a reliable preclinical model of depression, mimicking the onset and pathogenesis of depression in humans by transgenic, surgical, stress, and drug‐induced models.^7^ In particular, the CSDS model is a typical behavioral‐based preclinical tool inducing long‐term depressive‐ and anxiety‐like physiological and behavioral phenotypes in rodents.^8^, ^9^ The CSDS model leverages chronic stress exposure to induce a depression‐like state in experimental animals, simulating the exposure to stress in humans. Phenotypical alterations can be evaluated by assessing behavioral changes, as well as the extent of behavioral reversal during animal drug testing.^10^, ^11^, ^12^

Unfortunately, a high unmet need for an optimized CSDS experimental cage remains, given the lack of specification standardization in this regard.^13^ For instance, intra‐cage separation and build‐in blocks are subjected to lab‐to‐lab variation. Such a high variability may lead to additional challenges to experimental reproducibility between laboratories.^14^, ^15^, ^16^, ^17^ Currently, custom‐built devices can only be used for CSDS modeling with a single application scenario. Further, crowded living spaces also pose a challenge to lab animal welfare.^18^ Specifically, the leakage of conventional water bottles may potentially cause additional disturbance to the tested animals, undermining the authenticity of behavioral study results involving water bottles, i.e. the sucrose preference test (SPT).

To overcome above disadvantages, a novel rodent‐specific experimental cage system has thus been designed to support the optimization of the CSDS model. This system encompasses several distinct features. First, an empirical specification is made available to support test apparatus standardization and user‐friendliness. Second, the system design mimics the natural habitat of rodents. Third, the cages enable multiple usage scenarios, allowing for stress modeling and feeding. Fourth, the CSDS device has obtained patent authorization and can be widely applied in the field of scientific research. Notably, the incorporation of an S‐shaped drinking straw effectively mitigates water leakage during the sucrose preference test (SPT), as well as maintaining a dry cage environment to minimize disturbance to rodents.

## 2 METHODS

### 2.1 CSDS cage apparatus

**FIGURE 1:**
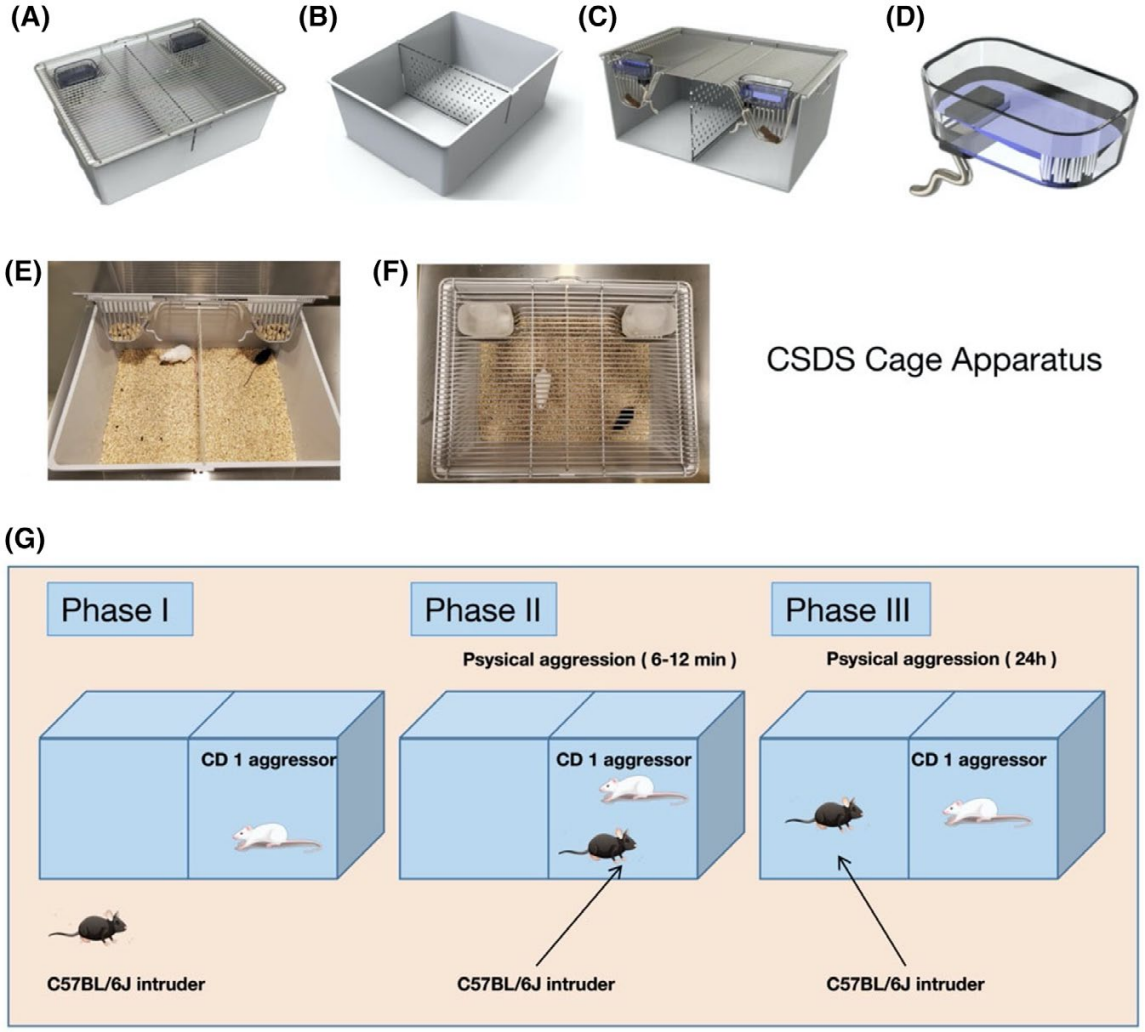
Design of CSDS cage apparatus and modeling paradigm. (A–F), Display images of innovative device designs. (G), Flow chart of CSDS modeling paradigm.

As shown in Figure 1A–F, a standardized experimental cage device for CSDS modeling was built with slots in the middle of the opposite side walls, and the two ends of the partition board were connected to the corresponding card slots. The body of the box was designed as a cuboid structure. A connecting plate was folded outward at the upper end of the box body. The cover also included a rectangular metal frame complementing the upper part of the box. The outer side was connected to a hook‐shaped handle linked to the rectangular frame sides by a rotating torsion spring. The corresponding connecting plate can be clamped tightly by rotating the torsion spring. The middle partition may be removed, allowing the cage to be used for experimental purposes and improving utilization efficiency.

In addition, an S‐shaped drinking pipe was linked to a water bottle equipped with a high‐temperature resistant rubber interface, which could be sterilized under high temperatures and effectively seal the drinking pipe (a shaped metal pipe). The shaped metal pipe was bent into multiple S‐shapes and extended to one side. A valve structure in the lower end of the spherical water delivery pipe was formed by making a cross shaped incision at the top of the spherical pipe, which could effectively prevent water leakage. The shaped metal pipe can be used with both rats and mice. The bottom of the cover was designed with pockets for carrying rat food and a for holding leak‐proof water bottles with S‐shaped drinking water pipes. The pocket was structured to be wider at the top and relatively narrow at the bottom, forming a funnel‐shaped structure. A partition board could be located in the middle of the box. The novel design of the partition board and accommodating slot allows a standardized living space for experimental mice and ensures the welfare for the tested rodents.

### 2.2 Animals and groups

For assessing the cages, 95 SPF‐grade healthy male C57BL/6J mice with body weight 20 ± 2 g and 80 SPF‐grade male CD1 mice with body weight 30 ± 2 g were used. Both strains were purchased from Beijing Vital River Laboratory Animal Technology Co., Ltd., license number: SCXK (Beijing) 2021–0006. The mice were housed in groups of five per cage with food and water ad lib and a 12‐h light cycle from 08:00–20:00. The animals were subject to a controlled temperature of 21 ± 2°C and humidity of 50 ± 10%. Prior to the test, the animals were acclimatized for 7 days. The mice were weighed to obtain baseline body weights and sucrose preference percentages were noted. Fifteen mice were used as the standard control group, and 65 mice were used as model mice. The animals were further subdivided into three groups according to the results of subsequent modeling, including a standard group, a model group, and a drug intervention group, with 15 mice in each group.15 mice were used as CD1 induced attack mice. C57BL/6J mice and CD1 mice are raised in different rooms with the same environmental conditions. All experimental operations were approved by the Institutional Committee for Animal Care and Use of Shandong University of Traditional Chinese Medicine (Approval ID: SDUTCM20190904013).

### 2.3 Treatments

The control group was given physiological saline gavage (10 mL kg^−1^ d^−1^) without any stress stimulation. The model group was given physiological saline gavage (10 mL kg^−1^ d^−1^). The Model+Fluoxetine group was given fluoxetine capsules by gavage (2.67 mg kg^−1^ d^−1^). Treatments were given to the groups for 14 days at 9:00 a.m. daily.^19^ Behavioral tests were performed separately 1 h after the last gavage to observe the effect of fluoxetine on improving depressive behavior.

### 2.4 CSDS paradigm

The experimental procedure for the social defeat model has been previously documented and is indicated in Figure 1G.^14^ In the first phase, the mice were acclimatized to the social defeat cage and developed territorial awareness. CD1 mice (i.e., the resident mice) were placed individually in side A of the social defeat cage and provided with adequate food for a period of 7 days. In the second phase, aggressive CD1 mice were screened and selected. C57BL/6J mice were used and placed in side A of the cage with the CD1 mice for three consecutive days, in order to induce aggressiveness in CD1 mice. Each induced CD1 aggressive mouse was assessed for aggressiveness. CD1 mice that exhibited aggressive behaviors for at least two successive days, showing an attack latency of no more than 60 s and made at least two attacks within 180 s were considered to be modeled. Unqualified CD1 mice were excluded. In the third phase (i.e. C57BL/6J mouse mimicry), the mimicked C57BL/6J mice were placed in the CD1 mouse residence and received aggressive CD1 attacks for 10 min. After 10 min, they were placed on the B side of the social defeat cage, where the mimicked C57BL/6J mice observed the CD1 mice through the middle transparent partition and were exposed to the scent of CD1 mice for 24 h. After that, the C57BL/6J mice were placed in another CD1 mouse cage. This was repeated for 10 days to ensure that a different CD1 mouse stress was applied daily to the C57BL/6J mice.

### 2.5 Phenotype detection

#### 2.5.1 Body weight status

Body weight is known to correlate with the status of depression.^20^ The mice were weighed before the experiments and after the modeling was completed.

#### 2.5.2 Sucrose preference test

The sucrose preference field was adopted to measure depressive‐like behaviors.^21^ Prior to the experiment, a 48‐h period of sucrose water training was carried out. For the first 24‐h, two bottles of 1% sucrose water were placed in the water intake pocket at each end of the cage; one bottle of 1% sucrose water and one bottle of pure water were placed in the water intake at each end of the cell in the second 24‐h period. The positions of the two bottles were exchanged at the 12th h to avoid position preference. After 24 h of water deprivation, each mouse was given one bottle of 1% sucrose water and one bottle of pure water. The positions of the two bottles were exchanged at the 12th h to avoid position preference. The amount of sucrose water and purified water consumed in 24 h was recorded. The extend of sucrose preference was calculated. The degree of pleasure deficit was assessed by detecting response of the mice to the reward. Sucrose preference (SP) = sugar‐water consumption (g)/[sugar‐water consumption (g) + water consumption (g)] × 100%. C57BL/6J mice (depression‐susceptible mice, about 65%) were screened according to the sugar water preference value, and unqualified C57BL/6J mice were excluded.

#### 2.5.3 Female urine sniffing test

The female urine sniffing test was utilized to assess reward‐seeking behavior.^22^ Sterile swabs were fixed on the side of the mouse cage 1 h before the experiment, and the mice were acclimated to the presence of the swabs. The light intensity of the laboratory was adjusted to 3 lux. The experiment was divided into three procedures. First, a swab dipped in pure water was placed on the side of the cage for 3 min. Second, the swab dipped in pure water was removed. Next, a swab dipped in fresh urine from a homozygous female (in estrus) was placed on the side of the cage after 45 min, and the sniffing time of the mice within 3 min was recorded. The amount of liquid dipped in the swab was 80 μL.

#### 2.5.4 Novelty‐suppressed feeding test

The novelty‐suppressed feeding (NSF) assay, measuring the latency to eat, was adopted to assess anxiety‐like behavior in the model.^23^ A white opaque resin test chamber with an open top was used, measuring 40 cm × 40 cm × 50 cm, and the bottom was covered with approximately 2 cm thick bedding, with six feed pellets placed in the center. After a 24‐h fast, the mice were placed in the same corner of the box and times to start sniffing the food were recorded, defined as the feeding latency. The test was conducted for 5 min, with the maximum feeding latency recorded as 5 min if the animal did not consume the food.

#### 2.5.5 Open field test

The open‐field test (OFT) was used to assess locomotion and anxiety‐like behavior in mice.^24^ The XR‐Super Maze video tracking analysis system conducted the behavioral test. The mice were placed in the central zone of the box (L × W × H = 800 cm × 800 cm × 50 cm). A camera and video synthesis system were used to record their activities. The total movement distance, mid‐zone distance, central zone entry times, central zone residence time, and other related parameters of each mouse in the box were analyzed over 6 min.

#### 2.5.6 Tail suspension test

The tail suspension test (TST) is widely used to evaluate depression‐like behavior in mice.^25^ One‐third of the mouse tail tip was attached to the tail suspension device with medical tape, and the mouse was suspended in an upright orientation, so that its head was facing the lens, about 30 cm from the ground, and its movements were recorded using the Smart 3.0 video tracking analysis system. The camera recorded the TST of each group of mice for 6 min, and the movements were recorded for 4 min after a 2 min acclimation. The immobility time was measured.

#### 2.5.7 Forced swimming test

The forced swim test is widely used to evaluate depression‐like behavior in mice.^25^ Each mouse was placed individually into a plexiglass cylinder (H: 40 cm, diameter: 12 cm) at a fixed water depth (30 cm, 23°C). In the training period, the mice were placed in the water for 15 min and then transferred to a dry environment (30°C, 30 min) . The formal experiment began after 24 h. Smart3.0 video tracking analysis system was used to collect and analyze swim data over 5 min. The suspension incubation period, time, and frequency were recorded, and after each mouse was taken out the water was changed and the cylinder wall cleaned. The experiment was conducted under a dark red light from 12:00 to 16:00. Mice that remained passively suspended with only slight movements to keep the nostrils above the water surface were judged to show immobile behavior.

### 2.6 Statistical analysis

All statistics in this study were analyzed using SPSS 22.0 software and expressed as mean ± SEM. The *t*‐test was used to compare the two groups, and one‐way ANOVA was used to compare more than two groups. The statistical difference threshold was designated at *p* < 0.05.

## 3 RESULTS

### 3.1 Body weight status

**FIGURE 2:**
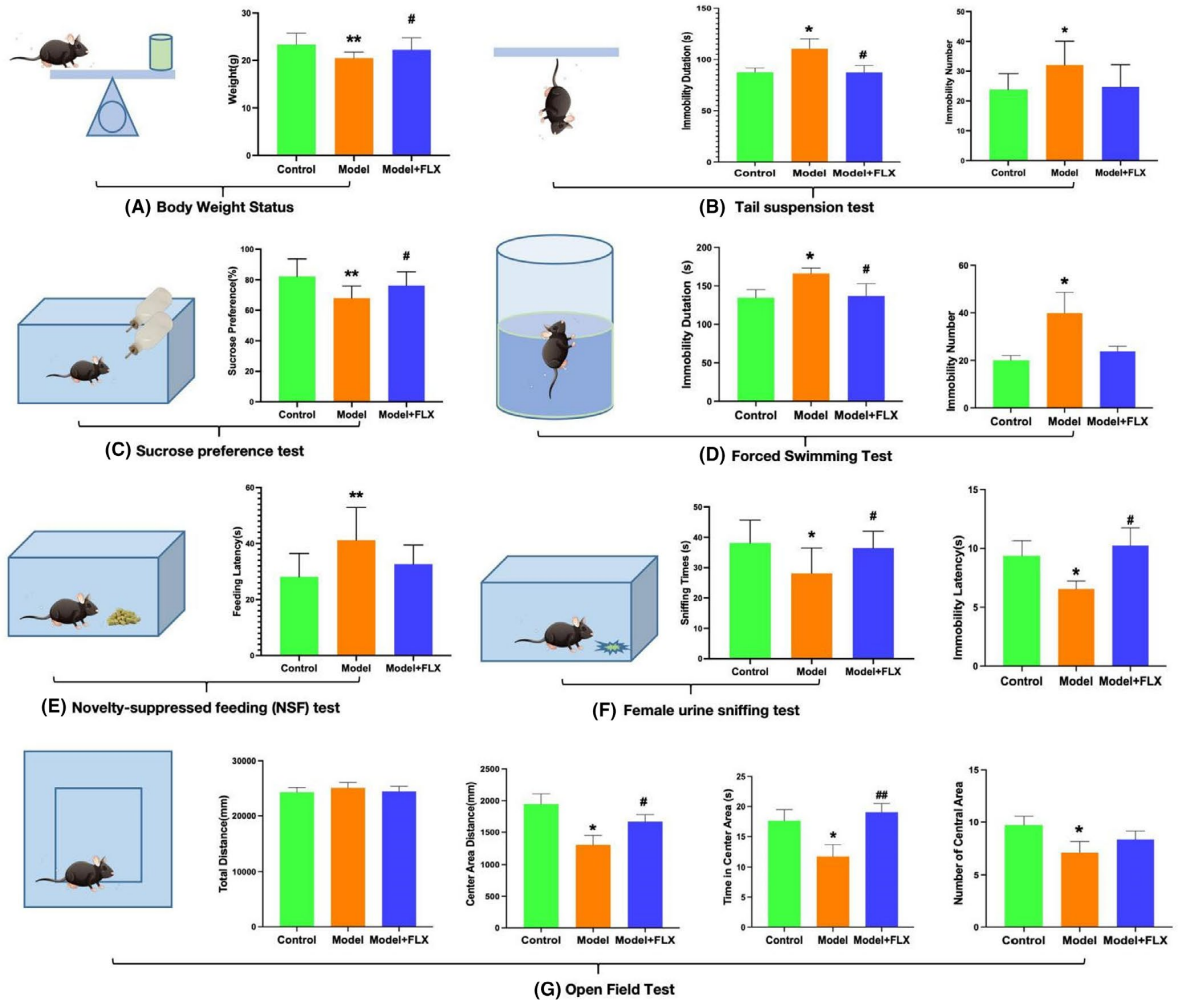
Novel devices for induction and evaluation of depression‐like emotion in rodents after chronic social defeat stress(CSDS). (A), Changes in mice body weight status after CSDS. (B–G), Results of behavioral tests. (B), Tail suspension test (TST): immobility number and immobility duration of TST. (C), Sucrose preference test (SPT): sucrose preference (%). (D), Forced swimming test (FST): immobility number, duration and latency of FST. (E), Novelty‐suppressed feeding (NSF) test: feeding latency(s). (F), Female urine sniffing test: sniffing times(s). (G), Open field test (OFT): total distance, central area distance, numbers of moving into the central area, time spent in the central area. *p < 0.05 compared to the control group, **p < 0.01 compared to the control group, ^#^ *p* < 0.05 compared to the model group, ^##^ *p* < 0.01 compared to the model group.

Following chronic social defeat stress (CSDS), the body mass of the CSDS‐exposed mice exhibited a significant reduction compared to the control group (*p* < 0.01), thereby confirming the successful establishment of the model. Additionally, the body mass of mice treated with fluoxetine demonstrated a significant increase relative to the model group (*p* < 0.05), suggesting that fluoxetine ameliorates the reduction in body mass observed in depressed mice. These findings are illustrated in Figure 2A.

### 3.2 Tail suspension test

As shown in Figure 2B, compared with the control group, the number and latency of immobility in CSDS mice were significantly increased (*p* < 0.05), indicating depressive‐like behaviors in the mice. Fluoxetine treatment significantly reversed the modeling effect and alleviated depressive‐like behavior in mice(*p* < 0.05).

### 3.3 Sucrose preference test

As shown in Figure 2C, compared with the control group, CSDS mice showed significantly decreased sucrose consumption (*p* < 0.01), indicating symptoms of loss of pleasure and depression. Compared with the model group, the fluoxetine treatment group showed a significant increase in glucose consumption rate in mice (*p* < 0.05).

### 3.4 Forced swimming test

As shown in Figure 2D, compared with the control group, the number and latency of immobility in CSDS mice were significantly increased (*p* < 0.05), indicating depressive‐like behavior in the mice. Fluoxetine treatment significantly reversed the modeling effect and alleviated depressive like behavior in mice (*p* < 0.05).

### 3.5 Novelty‐suppressed feeding test

As shown in Figure 2E, compared with the control group, the feeding latency of CSDS mice increased (*p* < 0.01); compared with the model group, the fluoxetine treatment group showed a decreasing trend in feeding latency.

### 3.6 Female urine sniffing test

As shown in Figure 2F, compared with the control group, the sniffing time of CSDS mice was significantly reduced (*p* < 0.05). Compared with the model group, the sniffing time of mice in the fluoxetine treatment group was significantly increased (*p* < 0.05), indicating that fluoxetine treatment successfully reversed the modeling effect.

### 3.7 Open field test

As shown in Figure 2G, no significant difference was found for the total distance moved by the mice among the groups. However, the distance moved in the central area by the model mice significantly decreased (*p* < 0.05).The time in the central area (*p* < 0.05) and the number of entries into the main area decreased significantly (*p* < 0.05).The results showed that the CSDS device did not affect the activity ability of the model mice, but could successfully induce emotional disorders (i.e. depression and anxiety‐like behavior). Fluoxetine treatment was found to alter mood‐related behaviors in mice.

## 4 DISCUSSION

The CSDS model successfully induced stress from a myriad of environmental factors, inducing depressive‐like behaviors in rodent.^26^, ^27^ Many epidemiological and clinical studies have confirmed that the etiology of depression is multifaceted and complex. It has a poor prognosis and is associated with a high suicide rate.^28^ Meanwhile, the need for a reliable and standardized preclinical model remains unmet and is crucial to support new drug development for depression.

In our study, in order to test the modeling effect of the apparatus, a variety of behavioral models were adopted to measure phenotypic changes in mice: body weight, locomotion, anxiety‐like behavior in the OFT, depression‐like behaviors in the TST, SPT, forced swimming test (FST), NSF, and female urine sniffing test. From the results, it was found that the model had consistently induced depressive‐like behaviors in mice across the tests. Weight loss in mice was also successfully induced and quantified in the CSDS modeling.

The sucrose preference value in the SPT decreased significantly, indicated a reduction of anhedonia‐like behavior. In the female urine sniffing test, the sniffing time of mice was significantly reduced. In the OFT, the reduced distance moved in the central area, the residence time in the central region, and the number of entries into the central area indicated depressive‐ and anxiety‐like behaviors. In the TST, the number of immobility and immobility times of the mice were significantly extended. In the FST, the induced swimming mice's numbers of suspension and the suspension immobility time was prolonged, indicating that the model mice had depressive and learned helplessness behavior after modeling. The results above support the success of the modeling effect under the CSDS model. Fluoxetine served as a positive control to reverse the modeling effect in the mice. The changes are compatible with existing CSDS model mouse studies.^29^, ^30^, ^31^, ^32^, ^33^

**FIGURE 3:**
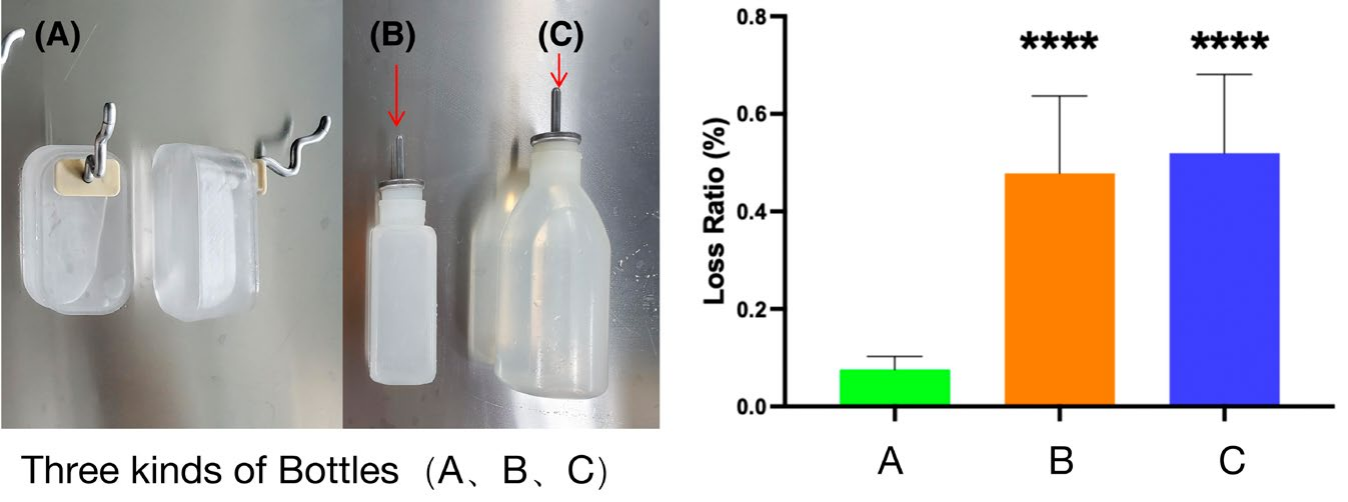
Water bottle comparison and leakage test, (A), Innovative Water Bottle; (B), Traditional Small Water Bottle; (C), Traditional Big Water Bottle. ****p < 0.001 compared to the control group.

The SPT is a commonly adopted behavioral test for assessing anhedonia and depressive‐like behavior in rodents, but a reliable measurement of sucrose water is still needed. In our apparatus, an S‐shaped drinking water pipe was introduced. To validate the design, a sucrose water test loss rate (i.e. water bottle leakage) test was performed, with precise measurement of the water before and after the experiments. The loss rate of spilled water during the release process is shown in Figure 3. The test includes six bottles each with 50%, 70%, and 90% of the water bottle capacity. Loss rate (%) = (pre‐experimental water bottle quantity − post‐experimental water bottle quantity)/pre‐experimental water bottle quantity. Compared to conventional small and big water bottles, the leak‐proof water bottle with the S‐shaped drinking pipe (i.e our innovative water bottle) showed a significant improvement in loss rate (****p < 0.0001), confirming the enhanced ability of the innovative water bottle to prevent water leakage.

## 5 INNOVATION AND LIMITATIONS

In the current study, a novel CSDS model apparatus was introduced and validated. The key features include an easy‐to‐use defeat cage, a flexible transparent partition for saving living space, as well as the S‐shaped leak‐proof water bottle design. In particular, the S‐shaped leak proof water bottle addressed existing challenges to the issue of water bottle leakage in conventional experiments, which contaminate the living space of the lab rodents, and more importantly improved the accuracy of water bottle‐related studies. Based on the existing apparatus prototype, research team would file a patent for the design, proceed to finalization of the CSDS model specification and commercialization.

## 6 CONCLUSION

A novel CSDS cage apparatus was successfully validated via a number of commonly used depressive‐like behavior tests in rodents, with several distinctive modifications including a user‐friendly defeat cage design, an adjustable intra‐cage transparent partition, and an S‐shaped leak‐proof water bottle design. The model also addressed the design challenges of conventional cages which may undermine the integrity of experimental results, providing an alternative promising option to researchers for preclinical behavioral studies.

## AUTHOR CONTRIBUTIONS

**Hao Zhang:** Conceptualization; data curation. **Dongmei Gao:** Funding acquisition. **Minghui Hu:** Data curation; resources. **Wanqing Zhou:** Investigation. **Muxuan Han:** Investigation. **Ya Sun:** Visualization. **Yang Zhang:** Resources. **Jieqiong Wang:** Resources; software. **Mingzhou Gao:** Methodology.

## FUNDING INFORMATION

This study was sponsored by the National Natural Science Foundation of China (No. 82204958, 82305065), Natural Science Foundation of Shandong Province (No: ZR2020ZD17), the Medical and Health Science and Technology Development Plan Project of Shandong Province (No.202105010467), the Traditional Chinese Medicine Science and Technology project of Shandong Province (No.Q‐2022059), and Traditional Chinese Medicine Emotional Disease and Brain Steady State Regulation Innovation Team (No. 2023KJ191).

## CONFLICT OF INTEREST STATEMENT

The authors declare that they have no conflicts of interest.

## ETHICS STATEMENT

All experimental operations were approved by the Institutional Committee for Anminal Care and Use of Shandong University of Traditional Chinese Medicine (SDUTCM20190904013).

## Data Availability

Data supporting this study's findings are available from the corresponding author upon reasonable request.
